# Analyzing Spanish News Frames on Twitter during COVID-19—A Network Study of El País and El Mundo

**DOI:** 10.3390/ijerph17155414

**Published:** 2020-07-28

**Authors:** Jingyuan Yu, Yanqin Lu, Juan Muñoz-Justicia

**Affiliations:** 1 Department of Social Psychology, Universitat Autònoma de Barcelona, 08193 Barcelona, Spain; Juan.Munoz@uab.cat; 2 School of Media and Communication, Bowling Green State University, Bowling Green, OH 43403, USA; ylu@bgsu.edu

**Keywords:** Twitter, news frame, network analysis, topic modeling, Spain

## Abstract

While COVID-19 is becoming one of the most severe public health crises in the twenty-first century, media coverage about this pandemic is getting more important than ever to make people informed. Drawing on data scraped from Twitter, this study aims to analyze and compare the news updates of two main Spanish newspapers *El País* and *El Mundo* during the pandemic. Throughout an automatic process of topic modeling and network analysis methods, this study identifies eight news frames for each newspaper’s Twitter account. Furthermore, the whole pandemic development process is split into three periods—the pre-crisis period, the lockdown period and the recovery period. The networks of the computed frames are visualized by these three segments. This paper contributes to the understanding of how Spanish news media cover public health crises on social media platforms.

## 1. Introduction

As COVID-19 is becoming a global health crisis, it has been announced as pandemic by World Health Organization (WHO, Geneva, Switzerland) on 11 March [1]. Three days after, being one of the most infected countries, Spanish prime minister Pedro Sanchez declared state of alarm. This is the second time that Spain declared a national lockdown, so the influence of the pandemic on Spain is substantial. As the situation of the pandemic became stable, the Spanish government announced a 4-step plan for the transition to a new normality on 3 May (*Plan para la transición hacia una nueva normalidad*), signaling that the pandemic is gradually becoming under control. 

News media are important information sources for the public during epidemic crisis [2], serving as interactive community bulletin boards, as well as global or reginal monitors [3]. With the prevalence of social media, news media organizations have been using these emerging tools to reach and engage boarder audiences during crises [4]. Twitter, being one of the most popular social media, has attracted a great number of traditional newspapers to digitalize real-time core information within 280 characters. While newspaper articles tend to use conflict, responsibility, consequence and savior frames in the coverage of epidemics, their Twitter accounts often post real-time updates, scientific evidence and actions [5]. The tones adopted in the two kinds of news are also different, with newspaper articles using more alarming and reassuring tones and Twitter updates using more neutral tones [5].

Scholars have been using the network analysis techniques to study news content. For example, Guo [6] proposed a Network Agenda Setting Model (NAS) to analyze the salience of the network relationships among objects and/or attributes. Inspired by this method, this study conducts network analysis on the Twitter posts, analyzing and comparing the news frames of the two most important general-interest and nationally-circulated Spanish newspapers (*El País* and *El Mundo*) during different stages of the COVID-19 crisis. The two selected newspapers are considered different regarding their political stance [7], with *El País* representing the political center-left media and *El Mundo* seen as a political center-right media outlet [8,9]. Discussion on the two media would allow us to better explore their particular news focus regarding their divergent political ideologies, thus illustrating a more comprehensive landscape of Spanish news coverage on the pandemic. Moreover, as this study focuses on the analysis of their Twitter content, compared with other newspapers, *El País* and *El Mundo* have the largest number of online followers, reflecting their substantial influence online. 

Two research gaps are filled in this paper. From the empirical approach, despite the fact that the two Spanish newspapers have been widely studied in the past epidemic crisis [10,11,12], their news posts on Twitter deserves more investigation in communication research. From the methodological perspective, manual coding process is generally applied in most of the network news agenda and news frame studies [13,14]. To enhance efficiency and minimize the biases involved in manual coding, this study combines unsupervised machine learning technique and network visualization method to make a fully automatic network study, which is a major methodological contribution to the news frame literature. 

## 2. Literature Review

### 2.1. Framing and Health Communication 

Framing is an important research focus in communication studies because how an issue is reported in news can influence how it is understood by audiences [15]. Entman [16] defined framing as “to select some aspects of a perceived reality and make them more salient in a communication text, in such a way as to promote a particular problem definition, causal interpretation, moral evaluation, and/or treatment recommendation for the item described” (p. 52). Frames in news media coverage can affect the topical focus and evaluative implications perceived by the audience, as well as their subsequent decision making about public policy [17]. 

News frames about health issues and diseases have been found to affect audiences’ understanding of health problems and their attitudes and behaviors [18,19]. Regarding the ongoing COVID-19 pandemic, the severity of the virus and preventive actions should be communicated to the public effectively. In this case, news media play an important role in enhancing public’s understanding of the highly contagious disease, as well as in influencing the attitudinal and behavioral response on the prevention, containment, treatment and recovery [18]. 

Empirical studies about news frames have been conducted during the past epidemic crisis. For example, Lee and Basnyat [18] focused on the news articles of Singaporean *Straits Times* during H1N1 pandemic and identified nine dominant frames via manual coding—*basic information*, *preventive information*, *treatment information*, *medical research*, *social context*, *economic context*, *political context*, *personal stories* and *other* (open-ended). Their study revealed that the news coverage focused more on H1N1 information updates and prevention than on other frames. In another one of their articles [20], four additional news themes were found—*imported disease*, *war/battle metaphors*, *social responsibility* and *lockdown policy*. Shih, Wijaya and Brossard [21] focused on news coverage about the mad cow disease, West Nile virus and avian flu from the *New York Times* by examining six frames—*consequence*, *uncertainty*, *action*, *reassurance*, *conflict*, *new evidence*. The results of their study revealed that the newspaper emphasized the consequence and action frames consistently across diseases but media concerns and journalists’ narrative considerations regarding epidemics did change across different phases of development and across diseases. 

### 2.2. Framing in Spanish News Media

According to the Association for Media Research (*Asociación para la Investigación de Medios de Comunicación*, http://reporting.aimc.es/index.html#/main/diarios), *El País* and *El Mundo* are the two most read general-interest newspapers in Spain in the first quarter of 2020. Comparative studies about these two newspapers have been conducted in various context. For example, Baumgartner and Chaqués-Bonafont [7] found that there are important news coverage differences between these two newspapers when they make explicit reference to individual political parties. Regarding negative news about corruption, *El País* tends to mention right-wing political party, while *El Mundo* mentions left-wing political party more often. The comparison between these newspapers in their news coverage about cannabis have also shown significant differences, *El País* focused more on the news about marijuana legalization, while *El Mundo* focused more on police and crime news on drug consumption [22].

During the Ebola outbreak, Ballester and Villafranca [12] studied the two newspapers together by comparing their news coverage of Ebola with other rare diseases. The word “terror” appears more frequently in Ebola related news, generating a higher level of anxiety toward Ebola than other diseases. Catalan-Matamoros et al. [10] studied the visual contents of the two newspapers, two main conclusions are made by the authors. First, the “conflict” frame dominates the portal of the two newspapers, which revealed alarming messages for the audience. Second, they found the total number of visual content increased rapidly in the first two days of the crisis and decreased from the fifth day. In sum, the authors described the first two days as “high risk phase” of the epidemic outbreak and from the fifth day onward the “less severe phase.” 

Regarding the ongoing COVID-19 crisis, researchers have found that there is a significant increase of coronavirus news in Spanish State of Alarm phase than the pre-alarm period and the total number of relevant news reported by *El Mundo* is much more than *El País* [23]. Thanks to the ease of information exchanges on social media platforms, Masip et al. [2] indicates that Spanish citizens are more informed during the coronavirus crisis than before. In this case, an in-depth analysis of social media news is warranted.

### 2.3. Methodological Background 

Latent Dirichlet Allocation (LDA) is frequently used to extract latent topics from large scale textual data and has also been widely applied for social media studies [24,25,26]. According to the developers of this technique, “LDA is a three-level hierarchical Bayesian model, in which each item of a collection is modeled as a finite mixture over an underlying set of topics. Each topic is, in turn, modeled as an infinite mixture over an underlying set of topic probabilities. In the context of text modeling, the topic probabilities provide an explicit representation of a document” [27] (p. 993).

Previous research has suggested LDA an appropriate method to study news media coverage [28]. For example, Heidenreich, Lind, Eberl and Boomgaarden [29] used this method to identify 16 frames from European refugee crisis news across five countries. For the COVID-19 related studies, Poirier et al. [30] applied LDA to identify six news frames (*Chinese outbreak, economic crisis, health crisis, helping Canadians, social impact, Western deterioration*) from 12 Canadian media sources.

In addition, network analysis methods have been widely adopted on communication studies. For example, regarding the mad cow disease, Lim, Berry and Lee [31] visualized the core word network of four groups (bureaucrats, citizens, scientists and interest groups) across four policy stages based on 6400 newspaper articles. They found the four groups focused on different policy issues and the news coverage did change over different stages. This study demonstrated that semantic network analysis is a powerful method for understanding issue framing in the policy process. Fu and Zhang [32] used word co-occurrence network to study NGOs’ HIV/AIDS discourse on social media and website. Their study revealed overlapping themes about HIV/AIDS across social media and website and NGOs use social media to engage with the government, as well as other health care resources. Kang et al. [33] examined the vaccine sentiment on Twitter by constructing and analyzing semantic networks of related information and found that semantic network of positive vaccine sentiment has a greater cohesiveness than the less-connected network of negative vaccine sentiment. This study sheds the light on discovering online information with a combination of natural language processing and network methods.

On the other side, Bail [34] conceptualized a method to combine natural language processing and network analysis to examine how advocacy organizations stimulate conversation on social media. The author’s idea is to convert the content of different documents into bag-of-words and then find the similarities (edges) between the documents by word co-occurrence. This method is further developed as a visualization tool to display a text network at group-word level [34,35]. In our case, each of the computed news frame (latent topic) is considered as a group of their relevant content, represented as nodes on the network and the edges between the frames are visualized according to the co-occurrence of the content and weighted by term frequency–inverse document frequency (tf-idf). To be clearer, the tf-idf is a numerical statistic to measure how relevant a word to a document in a corpus [36], it has been widely applied in text mining research, also in the abovementioned Bail’s work [34].

## 3. Methods

Our data are hydrated from open access institutional and news media tweet dataset for COVID-19 social science research [37], which includes the Twitter posts from the two selected Spanish newspapers from the end of February. The first step is data cleaning, in which all the retweets are removed. Then we deleted all the attached external website addresses, hashtags (#hashtags), mentions (@mentions), emojis, Arabic numbers and stopwords (e.g., prepositions, pronouns etc.), because such information is considered less meaningful in computational text analysis [38]. In addition, all the capital letters were converted to lower case (to standardize all the words) and we normalized the text with lemmatization (which refers to group together the inflected forms of a word) before the data are ready for the LDA model analyses.

Using the LDA function of R package “topicmodels” [39], we computed eight topics for each newspaper’s Twitter posts. The decision made on the number of topics is because too few topics make news frames less specific and too many topics make the network less interpretable [40]. In order to make the performance of the topic model more efficient, we used the Gibbs sampling method [39,41], one of the most widely used statistical sampling techniques for probabilistic models and short-text classification [42,43,44,45]. 

After having obtained the computed topics (news frames), we re-assigned each of the news tweets into their belonging frames, so we have a new dataset with the tweets of each newspaper categorized by the news frames. As the news focus regarding epidemics did change across different phases of the pandemic’s development [21], following the work of Pan and Meng that adopted a three-stage model to analyze news frames during a previous pandemic [46], we split each dataset by three periods. The pre-crisis period includes tweets before March 14 when Spanish national lockdown was announced. This is the period that the pandemic information has been reported but not been officially alarmed by Spanish government. The lockdown period includes tweets between 14 March and 11 May, the period that the Spanish government adopted a strict national confinement. The recovery period includes the tweets from 11 May (the day when Spain stepped into the first stage of social recovery) to 3 June (the last day of data collection). Finally, a network of relationships between news frames has been generated from their word co-occurrence matrix for each newspaper during each time period. Therefore, a total of six networks are constructed.

## 4. Results and Discussion

### 4.1. El País

For the *El País* dataset, a total number of 22,223 tweets are collected from 25 February 2020 to 3 June 2020. After removing retweets, 14,800 original tweets are saved for our in-depth analysis. Eight news frames have been successfully computed, they are “Livelihood” (family life and children), “Public Health Professional” (news about the department of public health), “Pandemic Update” (contagion and death poll), “Madrid” (news about Madrid), “Politics” (general political news), “State of Alarm” (Spanish government and PM’s announcement and policy update), “Economy” (the effect of the pandemic on Spanish economy) and “Covid Information” (general information about the pandemic). Table 1 presents the details of the eight news frames of *El País* with their top seven relevant words.

**Figure 1:**
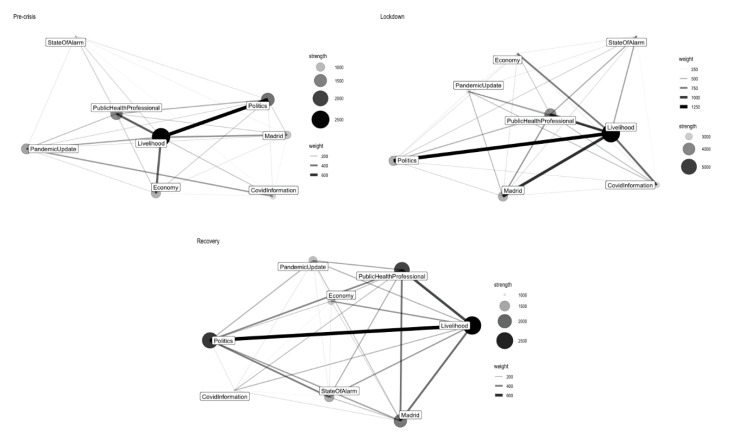
El País news frame network (from top to bottom and from left to right—pre-crisis period, lockdown period, recovery period).

Figure 1 presents the news frame network of the three segmented periods. Each of the nodes represents a news frame and the size of nodes indicates the strength of the node, also known as weighted node degree, it is the sum of the edge weights of the adjacent edges for each node [47], reflecting the importance of a node in a weighted network. The edges between the nodes represent the connection strength between two frames (normalized by tf-idf), it is the sum of the tf-idf value of the co-words. Table 2 presents the detailed information about the news frames in each of the three periods, with the node strength, number of tweets in each news frame and their proportion of the total number in each segment. Table 3 presents the table of the most weighted edges in the three time segments; it is able to provide us the news frames with the highest similarity ties. Overall, “Livelihood,” “Public Health Professional,” “Pandemic Update” and “Politics” are the most important news frames of *El País*. As the crisis is gradually under control, the “Pandemic Update” turned to be less prominent in the recovery period.

“Livelihood” is the most prominent news frame of *El País* and it shows a strong connection with “Politics,” “Economy” and “Public Health Professional” in the pre-crisis stage, suggesting a close connection with government policy and economic situation. In the next two periods, it started to have a more significant relation with “Madrid.” This is understandable because the Spanish capital suffered the most during the COVID-19 pandemic. According to the actual policy, the Community of Madrid is one of the last regions that stepped into the recovery plan [48] and this can also explain why the proportion of “Madrid” increased across the three time segments.

In addition, we indeed observed a news framing change in different stages of the pandemic outbreak. For example, the “Politics” frame is less reported in the second period while the “State of Alarm” and “Covid Information” frames have been paid higher attention during this stage. It is worth noting that although both frames have connections with others, no connections are observed between these two during the three periods, suggesting they are independent from each other. “State of Alarm” is a policy oriented news frame while “Covid Information” focused more on general sanitary information. 

As the crisis is gradually controlled, the pandemic related news frames (“Pandemic Update,” “State of Alarm,” “Public Health Professional” and “Covid Information”) are becoming less prominent in the recovery period. The media interests in general political news (“Politics”) decreased during the most difficult time but soon recovered with the crisis situation becoming stable. Regarding the network, the “Politics” frame has the strongest connection with “Livelihood” during all of the three periods. It also has significant relation with “Public Health Professional” (weight: 236.60) and “Economy” (weight: 154.96) during the pre-crisis period but the two connections have been developing in different trends. While “Politics” and “Public Health Professional” remained connected in the other two periods, the connection between “Politics” and “Economy” turned to be less significant. Instead, the “Politics” frame becomes more connected with “State of Alarm” and “Madrid.”

### 4.2. El Mundo

For the *El Mundo* dataset, a total number of 17,577 tweets are collected from 19 February 2020 to 3 June 2020. After removing retweets, 14,290 original tweets are saved for our in-depth analysis. Eight news frames are computed, six of which are considered the same as *El País*. They are “Madrid,” “State of Alarm,” “Covid information,” “Economy,” “Pandemic Update,” “Politics.” The two unique *El Mundo* frames are “Lockdown” (news about the confinement) and “Hospital” (news related to hospital, doctor and patient). Table 4 presents the news frames with their most relevant keywords.

**Figure 2:**
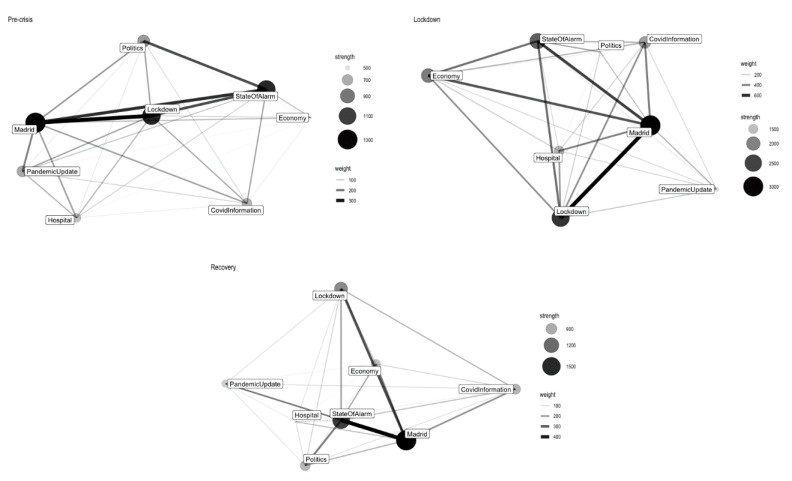
El Mundo news frame network (from top to bottom and from left to right: pre-crisis period, lockdown period, recovery period).

Figure 2 presents the network of the three segmented periods, Table 5 provides the detailed information of the news frames across time and Table 6 presents the detailed information of the most weighted edges. Generally speaking, “Madrid,” “State of Alarm” and “Lockdown” are the three most prominent news frames during the pre-crisis period, along with the crisis becoming more severe, “Covid Information” is paid more attention by the newspaper. And finally these four frames are the most prominent news frames during the recovery period.

“Madrid” is the most prominent news frame of *El Mundo* of all the time. The proportion of this topic is greatly changed from the second period to the third. As we have explained in the previous section, Madrid is the last region that stepped into recovery plan, so this change is understandable. The “Madrid” frame has the strongest connection strength with “Lockdown” and “State of Alarm” during the first two periods and the association between “Madrid” and “Covid Information” becomes more and more eye-catching during the last two periods. The second most important news frame is “State of Alarm,” it has been paid less attention during the lockdown period but still, shared a significant proportion of the total news coverage. The “State of Alarm” frame has the highest connection strength with “Madrid” and “Lockdown,” similar to “Madrid,” the relation between “State of Alarm” and “Covid Information” is becoming stronger during the second and third time segments (weight in the 2nd period: 259.72, in the 3rd period: 139.42). 

As Spain started to get recovered from the strict national lockdown, the proportion of the relevant news frames “Lockdown” and “Hospital” decrease during the recovery period but the connection between these two topics have been strengthened in this stage. As the “Lockdown” frame is highly associated with “Madrid” and “State of Alarm,” we assume this frame is strongly policy orientated. On the other hand, the “Hospital” frame includes both health and social news, so it is naturally associated the most with “Madrid” and “Lockdown.” Regarding the “Economy” frame, the proportion of this topic arrived its peak at the second period. It is significantly different from the frame “Politics,” which has been less adopted during the same period. Both of them have strong ties with “Madrid” and “State of Alarm” but no significant connections have been exposed between these two frames. 

Given that the frames “Covid Information” and “Pandemic Update” have almost no proportion changes during the three time periods, these two news frames are considered as stable news frames, tweets about “Pandemic Update” is slightly fewer than “Covid Information.” Regarding the network, like many other *El Mundo* news frames, both of the two have the strongest connection with “Madrid” and the tie between these two frames is getting more and more meaningful over time.

### 4.3. Comparative Discussion

Significant differences are observed between *El País* (EP) and *El Mundo* (EM) in the frames used in their Twitter news posts. First, the most prominent news frame of the two Spanish newspapers are different. While EP focused on “Livelihood,” EM tended to adopt the “Madrid” frame most frequently. Despite the fact that “Madrid” is also a frame in the EP dataset, it is considered as a peripherical news frame. Both of the two frames have the strongest connections with other topics in the networks, so these two frames can be seen as the motor themes of their newspapers on Twitter.

Second, both of the newspapers have two unique news frames. While the EP news coverage on Twitter focuses on “Livelihood” and “Public Health Professional,” we observed the “Lockdown” and “Hospital” frames in the EM Twitter posts. The “Livelihood” frame is somewhat similar to “Hospital,” because both of the two news frames contain social and living attributes. Nevertheless, their connection strength with the other common frames are different. While “Livelihood” associates the most with “Politics” and “Public Health Professional” in the EP networks, “Hospital” associates the most with “Madrid” and “Lockdown” in the EM networks. A possible interpretation of this difference is “Livelihood” is linked to government (including relevant government departments) policy but “Hospital” is more linked to the news about specific regions. Also, EP shows higher attention to the Ministry of Health and professional perspective by adopting the “Public Health Professional” frame while EM focuses more on the effect of confinement from social perspectives with the “Lockdown” frame.

Third, although there are six common news frames identified in the Twitter posts of both newspapers but the longitudinal changes in their proportion over time are different. For example, the “Economy” related news tweets are increasingly scarce over time in the EP dataset but for EM, such information is more posted during the second time period (the lockdown period). Another significant example can be seen from the “Politics” frame. The EP Twitter account posted more politics-related news during the recovery period than during the lockdown period. But for EM, the increasing trend during the same periods is not so salient as EP. 

“State of Alarm” is the second most important news frame for EM on Twitter but this frame is not so prominent in EP Twitter posts. Although the most relevant keywords of this frame in the two datasets are almost the same but the connections are different in the networks. During the first two periods, “State of Alarm” is considered most associated with “Lockdown” and “Madrid” in the EM network, while it is mostly linked to “Livelihood” and “Public Health Professional” in the EP network. During the recovery period, the link between “State of Alarm” and “Politics” is strengthened in EP network, while the connection between “State of Alarm” and “Covid Information” is more eye-catching in the EM network. This finding implies that, with the pandemic crisis getting under control, Twitter posts about “State of Alarm” is more related to political news on EP but connected to health news more closely in the EM Twitter coverage.

## 5. Conclusions

This study analyzed and compared the frames of Twitter news posts in the two most important Spanish newspapers during Covid-19 pandemic crisis. With a combination of topic modeling and network analysis method, a general landscape of the news coverage of the two newspapers has been illustrated. We found that the center-left media focused the most on family life and living issues (“Livelihood”), while the center-right media focused the most on the Spanish capital news (“Madrid”). From the distribution and proportion of news frames, it can be concluded that *El País* focused the most on public health professionals and real-time alarming (“Pandemic Update”) information during the first two periods. The *El Mundo* coverage on Twitter focused on the state of alarm and confinement (“Lockdown”) related information. During the recovery period, the proportion of general political news (“Politics”) update is largely increased in *El País*, being the third most prominent news frame in this stage. Nevertheless, no such changes are observed in the results of *El Mundo*. Our results are consistent with the thesis proposed by Shih et al. [21] that media coverage about epidemics did change across different phrases of the crises. Given our limited data collection timespan and the unique characteristics of Twitter data, a more comprehensive analysis is needed for future studies. 

From the methodological approach, our method combination provides a dynamic overview of news frames’ evolution over time. The weighted node degree and the most weighted edges in each of the stages have been reported. Each of the motor themes (“Livelihood” for *El País*, “Madrid” for *El Mundo*) is the leading topics of all of the three time segments. Given the strong connections of the two topics with other frames, we observed a more unbalanced network structure in *El Mundo* dataset. Specifically, a second-level community is identified, which consisted of “Madrid,” “Lockdown” and “State of Alarm” in the pre-crisis period. The community is enlarged with “Covid Information” included in the last two periods. It implies that the content of the four news frames have a high degree of co-occurrence, they are relatively more independent from other frames. But the second-level community cannot be clearly observed in the *El País* network, thus, we believe that the news frames of *El Mundo* is more centralized than *El País*. 

Finally, several limitations of our study should be mentioned. First, previous literature has indicated that Twitter based short-text news updates are different from their full length articles [5]. In this case, it is worth noting that our results are solely based on the Twitter posts, which may not be generalized to the comparison between the contents of the two newspapers’ articles. Second, as the news coverage may less focus on the health issue in the pre-crisis period than in later stages and our adopted topic modeling method is highly depended on the vast dataset, the number of tweets in the pre-crisis period is much less than the two other periods, news frames on the first period may not be perfectly classified. Finally, although we have analyzed the two most important Spanish newspapers with different political stances, the number of research objects are still limited and we would like to include more newspapers and use a larger dataset as our improvement strategies for the future. 

## Figures and Tables

**Table 1:** El País Twitter news frames with most relevant words (translated into English).

**Livelihood**	**Public Health** **Professional**	**Pandemic Update**	**Madrid**
years	simon	spain	madrid
life	public	country	pass
live	mask	death	week
child	fernando	hour	confinement
leave	change	contagion	exit
son/daughter	should	die	common
family	form	data	phase
**Politics**	**State of Alarm**	**Economy**	**Covid Information**
police	government	crisis	person
think	sanchez	million	pandemic
inform	doctor	month	sanitary
question	minister	arrive	world
politics	alarm	spanish	virus
video	health	work	hospital
ask	president	economy	covid

**Table 2:** Detailed information of the (El País) news frame in each of the period (S: node strength. N: number of tweets. P: proportion.).

News Frames	Pre-Crisis Period			Lockdown Period			Recovery Period		
	S	N	P	S	N	P	S	N	P
Livelihood	2583.42	633	26.3%	5907.56	1625	18.1%	2808.26	618	18.4%
Public Health Professional	1414.49	356	14.8%	3857.47	1320	14.7%	2253.56	554	16.5%
Pandemic Update	1191.59	320	13.3%	2694.64	1226	13.6%	1317.59	368	11.0%
Madrid	915.14	206	8.6%	3436.31	1050	11.7%	1858.97	447	13.3%
Politics	1611.72	306	12.7%	3450.10	945	10.5%	2352.80	470	14.0%
State of Alarm	620.85	185	7.7%	2623.05	1107	12.3%	1452.55	389	11.6%
Economy	1087.55	229	9.5%	2631.96	791	8.8%	1199.97	265	7.9%
Covid Information	744.08	169	7.0%	2826.89	907	10.1%	996.43	249	7.4%

**Table 3:** Detailed information of the (El País) most weighted edges.

Pre-Crisis Period		Lockdown Period		Recovery Period	
Edge name	Edge weight	Edge name	Edge weight	Edge name	Edge weight
Livelihood–Politics	766.78	Livelihood–Politics	1273.50	Livelihood–Politics	738.83
Livelihood–Economy	468.20	Livelihood–Madrid	1052.63	Livelihood–Public Health Professional	536.00
Livelihood–Public Health Professional	463.99	Livelihood–Public Health Professional	956.40	Livelihood–Madrid	431.43
Livelihood–Madrid	321.80	Livelihood–Covid Information	800.83	Madrid–Public Health Professional	405.91
Covid Information–Pandemic Update	290.59	Livelihood–Economy	728.75	Politics–State of Alarm	386.56

**Table 4:** El Mundo Twitter news frames with most relevant words (translated into English).

**Madrid**	**State of Alarm**	**Lockdown**	**Covid Information**
madrid	government	confinement	world
pass	sanchez	person	country
common	alarm	doctor	pandemic
phase	pedro	leave	inform
health	ask	social	virus
week	president	secure/insurance	china
de-escalation	announcement	quarantine	port
**Economy**	**Pandemic Update**	**Hospital**	**Politics**
sanitary	spain	years	minister
crisis	death	hospital	police
million	case	death	iglesias
spanish	covid	patient	pablo
mask	contagion	doctor	investigation
economy	die	resident	press
euro	italy	child	civil

**Table 5:** Detailed information of the (El Mundo) news frames in each of the period. (S: node strength. N: number of tweets. P: proportion.).

News Frames	Pre-Crisis Period			Lockdown Period			Recovery Period		
	S	N	P	S	N	P	S	N	P
Madrid	1339.36	537	19.4%	3073.28	1389	17.6%	1713.88	694	21.9%
State of Alarm	1174.63	450	16.2%	2274.25	1126	14.2%	1407.71	542	17.1%
Lockdown	1177.45	426	15.4%	2616.23	1210	15.3%	1058.93	391	12.3%
Covid Information	688.13	319	11.5%	1760.66	958	12.1%	856.27	373	11.8%
Economy	482.75	200	7.2%	2039.09	927	11.7%	797.00	299	9.4%
Pandemic Update	714.74	296	10.7%	1212.13	806	10.2%	725.14	316	10.0%
Hospital	619.37	271	9.8%	1562.08	871	11.0%	615.52	245	7.7%
Politics	768.16	271	9.8%	1164.04	622	7.9%	846.89	308	9.7%

**Table 6:** Detailed information of the (El Mundo) most weighted edges.

Pre-Crisis Period		Lockdown Period		Recovery Period	
Edge name	Edge weight	Edge name	Edge weight	Edge name	Edge weight
Madrid–Lockdown	339.38	Madrid–Lockdown	744.75	Madrid–State of Alarm	455.67
Madrid–State of Alarm	276.40	Madrid–State of Alarm	560.53	Madrid–Lockdown	314.04
Lockdown–State of Alarm	240.89	Madrid–Economy	502.84	Politics–State of Alarm	244.29
Politics–State of Alarm	239.43	Lockdown–State of Alarm	436.85	Madrid–Economy	229.17
Madrid–Pandemic Update	178.53	Economy–State of Alarm	429.68	Madrid–Pandemic Update	214.04
